# Supplementary material for: CTC1-STN1 terminates telomerase while STN1-TEN1 enables C-strand synthesis during telomere replication in colon cancer cells
Source: Nat Commun. 2018 Jul 19;9:2827. doi: 10.1038/s41467-018-05154-z (PMC6053418; doi:10.1038/s41467-018-05154-z)
Supplement: Supplementary file 1 — Supplementary Information [file 41467_2018_5154_MOESM1_ESM.pdf]

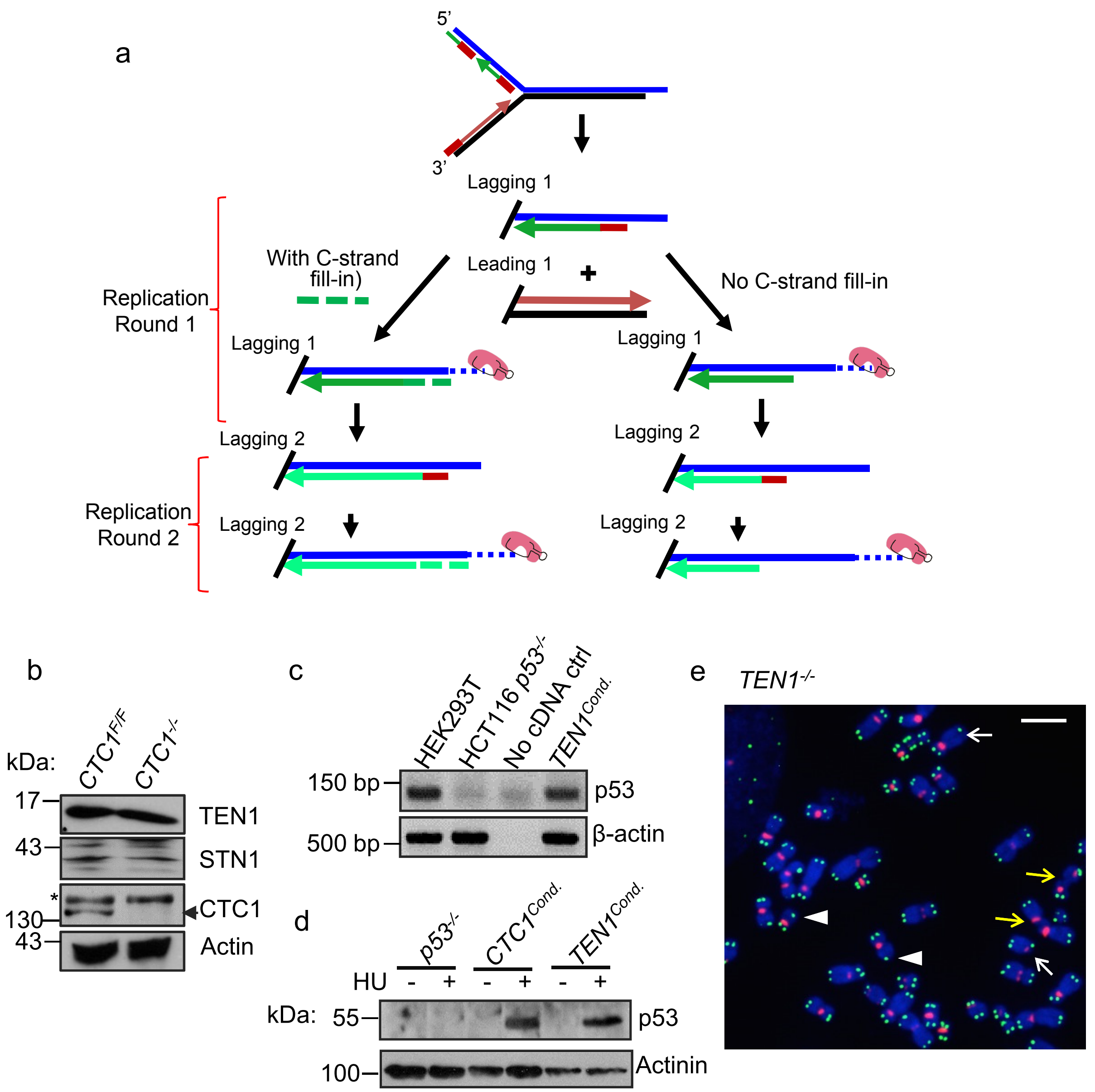

a
5'
3'
Lagging 1
With C-strand fill-in)
Leading 1
No C-strand fill-in
Replication Round 1
Lagging 1
Lagging 1
Lagging 2
Lagging 2
Replication Round 2
Lagging 2
Lagging 2
b
kDa:
CTC1F/F
CTC1-/-
17
TEN1
43
STN1
*
CTC1
130
43
Actin
c
HEK293T
HCT116 p53-/-
No cDNA ctrl
TEN1Cond.
150 bp
p53
500 bp
β-actin
d
p53-/-
CTC1Cond.
TEN1Cond.
HU
- + - + - +
kDa: 55
p53
100
Actinin
e
TEN1-/-

Supplementary Figure 1. TEN1 disruption causes a modest increase in telomere aberrations. (a) Cartoon illustrating how loss of C-strand fill-in leads to shortening of lagging strand telomeres following each round of replication. (b) Western blot showing expression level of TEN1, STN1 and CTC1 in *CTC1*$^{-/-}$ cells. CTC1 conditional cells were grown with/without tamoxifen for 7 days. Blot was probed with antibody to TEN1, CTC1, STN1 or actinin as a loading control. *, non-specific band. (c) RT-PCR showing p53 mRNA level in TEN1 conditional cells. HEK293T cells served as a positive control and HCT116 *p53*$^{-/-}$ cell as negative control. (d) Western blot showing p53 expression in CTC1 conditional and TEN1 conditional cells with/without HU treatment (2 mM for 24 hrs) to induce DNA damage. *p53*$^{-/-}$ HCT116 cell served as a negative control. (e) Representative metaphase spread from *TEN1*$^{-/-}$ cells after 10 days growth with tamoxifen. Chromosomes were hybridized with $(C_3TA_2)_3$ telomere probe (green) and centromere probe (red) and stained with DAPI (blue). White arrows indicate partial or full telomere loss (signal free ends, SFE). White arrowheads indicate sister chromatid association. Yellow arrows indicate chromosome fusions. Scale bar indicates 5 μm.

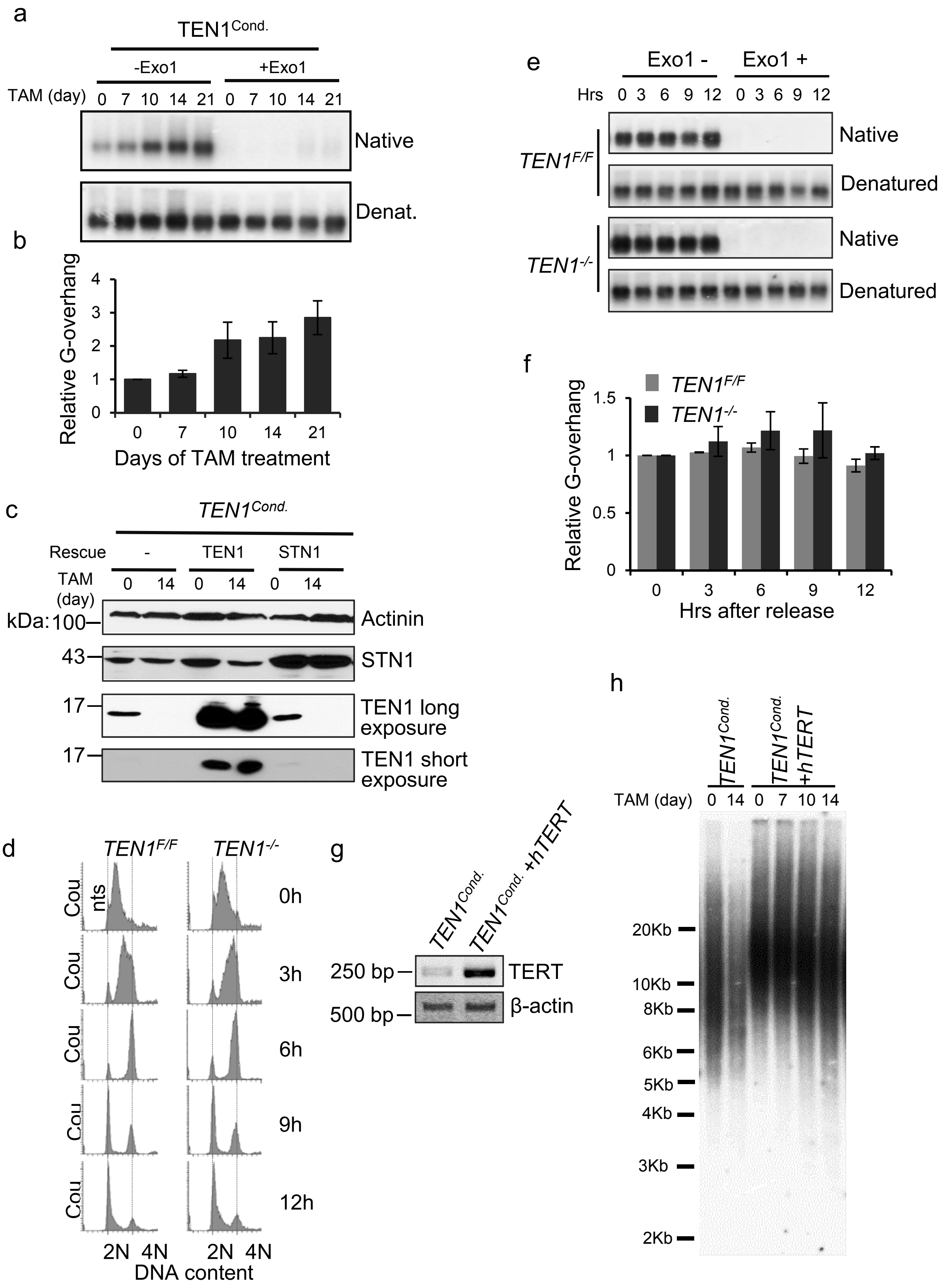
a
TEN1Cond.
-Exo1
+Exo1
TAM (day)
0 7 10 14 21 0 7 10 14 21
Native
Denat.
b
Relative G-overhang
0 7 10 14 21
Days of TAM treatment
c
TEN1Cond.
Rescue
-
TEN1
STN1
TAM (day)
0 14 0 14 0 14
kDa: 100
43
17
17
Actinin
STN1
TEN1 long exposure
TEN1 short exposure
d
TEN1F/F
TEN1-/-
Counts
0h
3h
6h
9h
12h
2N 4N 2N 4N
DNA content
e
Exo1 -
Exo1 +
Hrs
0 3 6 9 12 0 3 6 9 12
TEN1F/F
TEN1-/-
Native
Denatured
Native
Denatured
f
TEN1F/F
TEN1-/-
Relative G-overhang
0 3 6 9 12
Hrs after release
g
TEN1Cond.
TEN1Cond. +hTERT
250 bp
500 bp
TERT
β-actin
h
TEN1Cond.
TEN1Cond. +hTERT
TAM (day)
0 14 0 7 10 14
20Kb
10Kb
8Kb
6Kb
5Kb
4Kb
3Kb
2Kb

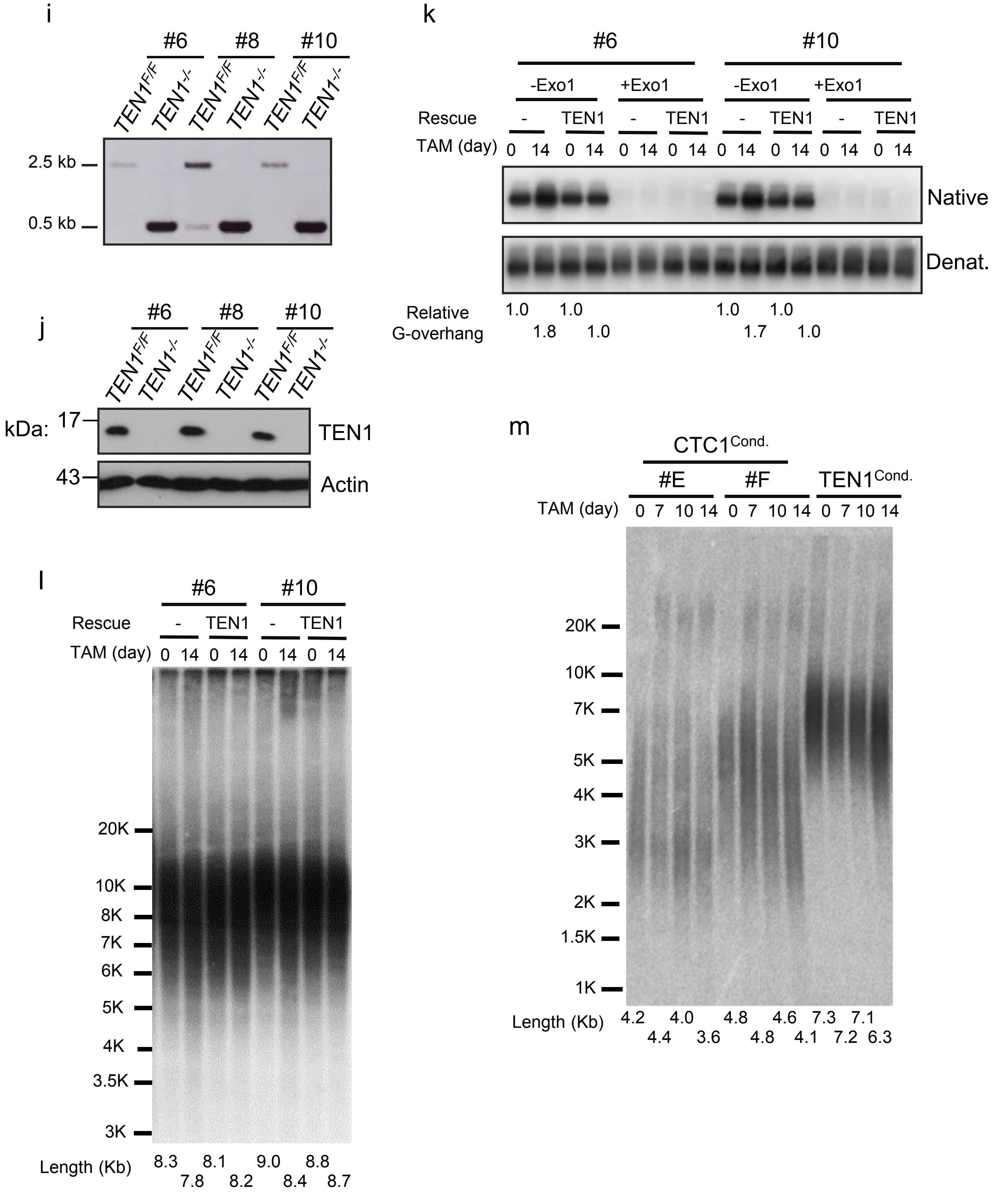
i
#6
#8
#10
TEN1F/F
TEN1-/-
2.5 kb
0.5 kb
j
kDa:
17
43
TEN1
Actin
k
-Exo1
+Exo1
Rescue
TAM (day)
Native
Denat.
Relative
G-overhang
1.0 1.0 1.0 1.0
1.8 1.0 1.7 1.0
l
TEN1
20K
10K
8K
7K
6K
5K
4K
3.5K
3K
Length (Kb)
8.3 8.1 9.0 8.8
7.8 8.2 8.4 8.7
m
CTC1Cond.
#E
#F
TEN1Cond.
1.5K
2K
1K
4.2 4.0 4.8 4.6 7.3 7.1
4.4 3.6 4.8 4.1 7.2 6.3

Supplementary Figure 2. Changes in relative G-overhang length during the cell cycle. (a-b) G-overhang abundance in TEN1 conditional cells treated with/without tamoxifen for the indicated times. (a) Gels showing hybridization of $TAA(C_3TA_2)_3$ probe to genomic DNA under native and denaturing conditions. (b) Quantification of relative G-overhang abundance (Error bars, mean $\pm$ S.E.M., n = 3 exp.). (c) Western blot showing overexpression of FLAG-TEN1 or FLAG-STN1 rescuing alleles in *TEN1*$^{-/-}$ cells. Loading control is actinin. (d-e) Overhang signal from synchronized TEN1 conditional cells after release from thymidine block. (d) FACS data showing DNA content and synchrony of cells used for (e). (e) Representative gels showing overhang signal in *TEN1*$^{F/F}$ or *TEN1*$^{-/-}$ cells. DNA was hybridized with $TAA(C_3TA_2)_3$ probe before and after denaturation. (f) Quantification of overhang signal (Error bars, mean $\pm$ SEM, n = 3 exp.). (g) RT-PCR showing hTERT overexpression. (h) Southern blot showing increase in terminal restriction fragment length in TEN1 conditional cells after hTERT overexpression. hTERT expressing cells were harvested for TRF analysis ~4 weeks after transfection with hTERT expression construct. i-m) Analysis of multiple clones showing effects of TEN1 or CTC1 gene disruption on telomere length or G-overhang length**.** Clones were isolated after introduction of Cre-ER into TEN1$^{F/F}$ or CTC1$^{F/F}$ cells. (i) PCR verifying gene disruption in three additional TEN1 conditional clones (*TEN1*$^{F/F}$ cells expressing Cre-ER) after growth with tamoxifen for 7 days (*TEN1*$^{-/-}$). (j) Western blot showing levels of TEN1 in same cells as (I). Blot was probed with antibody to TEN1 or actinin as loading control. (k) G-overhang length in TEN1 conditional clones #6 and #10. Rescue: an exogenous TEN1 rescuing allele was introduced in to the TEN1$^{F/F}$ cells. G-overhang abundance was analyzed by in-gel hybridization following the indicated times of tamoxifen (TAM) treatment. Relative G-overhang abundance is shown below each lane. The signal from the TEN1$^{F/F}$ cells (TAM day 0) is set to 1. (l-m) Southern blots showing terminal restriction fragments in two additional TEN1 (l) or CTC1 (m) conditional clones treated with tamoxifen for the indicated times. Rescue: indicates introduction of exogenous TEN1 rescuing allele. Mean telomere length is indicated below each lane.

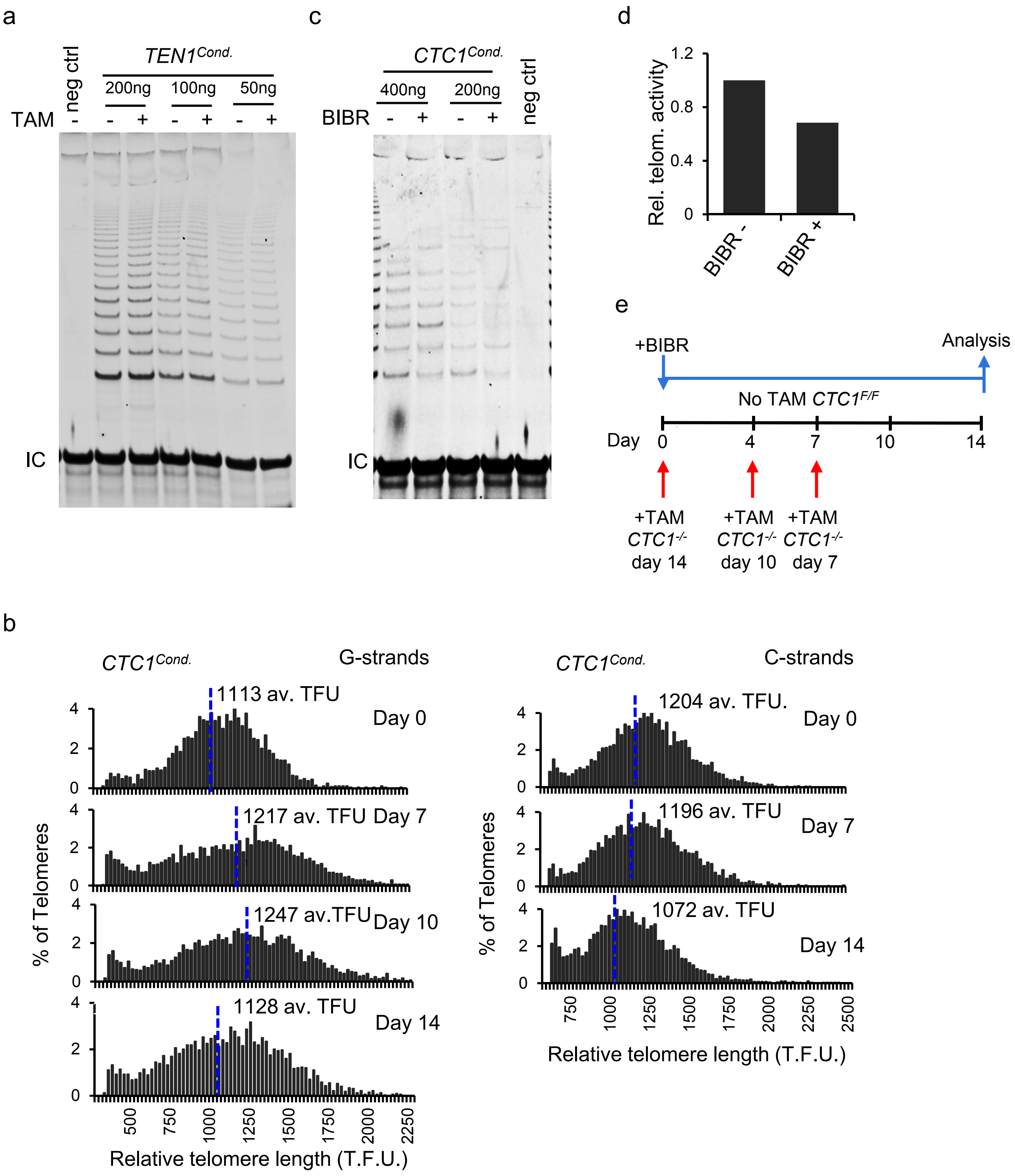
a
TEN1Cond.
neg ctrl
200ng
100ng
50ng
TAM - - + - + - +
IC
c
CTC1Cond.
400ng
200ng
neg ctrl
BIBR - + - +
IC
d
Rel. telom. activity
1.2
0.8
0.4
0
BIBR -
BIBR +
e
+BIBR
Analysis
No TAM CTC1F/F
Day 0 4 7 10 14
+TAM CTC1-/- day 14
+TAM CTC1-/- day 10
+TAM CTC1-/- day 7
b
CTC1Cond.
G-strands
1113 av. TFU
Day 0
1217 av. TFU
Day 7
1247 av.TFU
Day 10
1128 av. TFU
Day 14
% of Telomeres
500 750 1000 1250 1500 1750 2000 2250
Relative telomere length (T.F.U.)
CTC1Cond.
C-strands
1204 av. TFU.
Day 0
1196 av. TFU
Day 7
1072 av. TFU
Day 14
% of Telomeres
750 1000 1250 1500 1750 2000 2250 2500
Relative telomere length (T.F.U.)

Supplementary Figure 3. Telomerase levels and telomere length in *TEN1*$^{-/-}$ or *CTC1*$^{-/-}$ cells. (a) TRAP assay showing telomerase activity in TEN1 conditional cells after 14 days with/without tamoxifen. (b) Analysis of telomere length in *CTC1*$^{-/-}$ cells by Q-FISH. Cells were treated with tamoxifen for the indicated times. Metaphase spreads were hybridized with $(C_3TA_2)_3$ G-strand probe (left) or $(G_3AT_2)_3$ C-strand probe (right). Histograms show the distribution of relative telomere lengths expressed as fluorescence intensity (TFU, telomere fluorescence unit). A minimum intensity of 100 TFU was set as the cut-off. av.; median value, >2000 telomeres were quantified for each sample. (c) TRAP assay showing telomerase activity in *CTC1*$^{-/-}$ cells with or without 14 days of BIBR 1532 treatment. (d) Quantification of telomerase activity in (c). (e) Time-line for BIBR experiment shown in Figure 3.

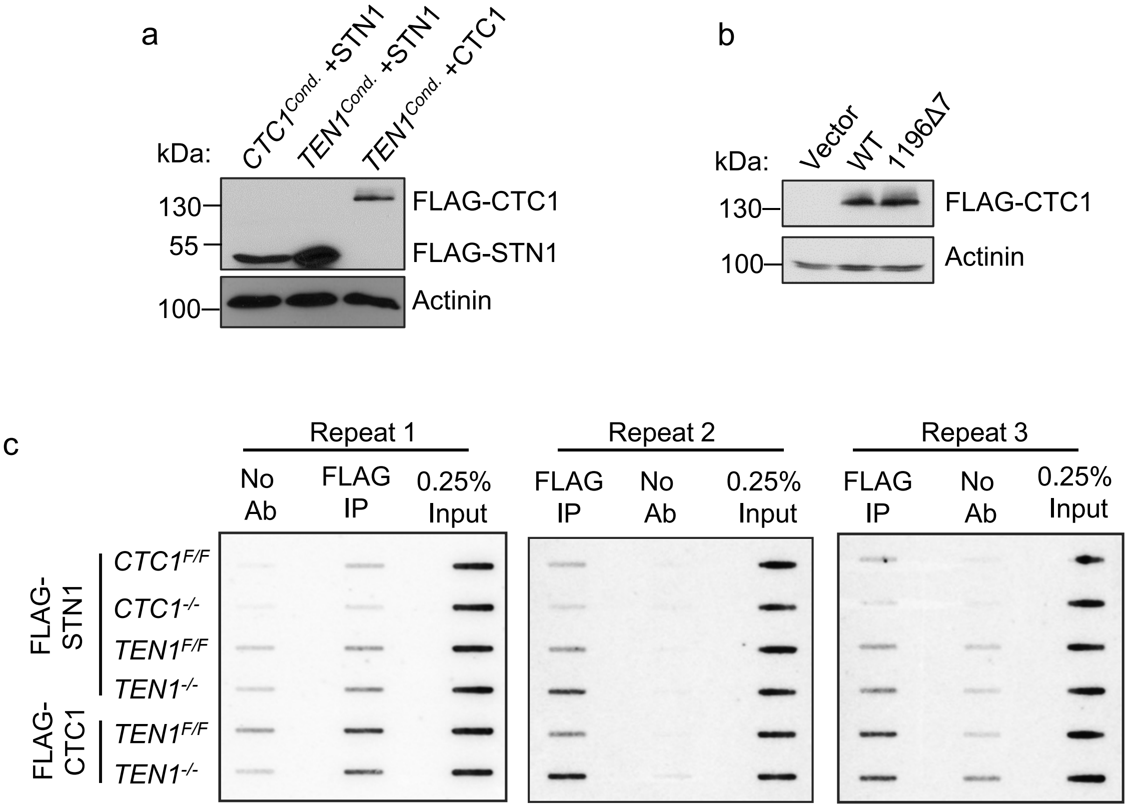

Supplementary Figure 4. Western blots showing levels of CTC1 and STN1 expression. (a) Western blot showing level of FLAG-STN1 or FLAG-CTC1 in *CTC1*$^{F/F}$ or *TEN1*$^{F/F}$ cells. Actinin is used as a loading control. (b) Western blot showing level of FLAG-CTC1 wild type and 1196Δ7 mutation in *CTC1*$^{F/F}$ cells. (c) Three repeats of slot blots to quantify FLAG-STN1 or FLAG-CTC1 localization to telomeres. Cells were grown with/without tamoxifen for 7 days.

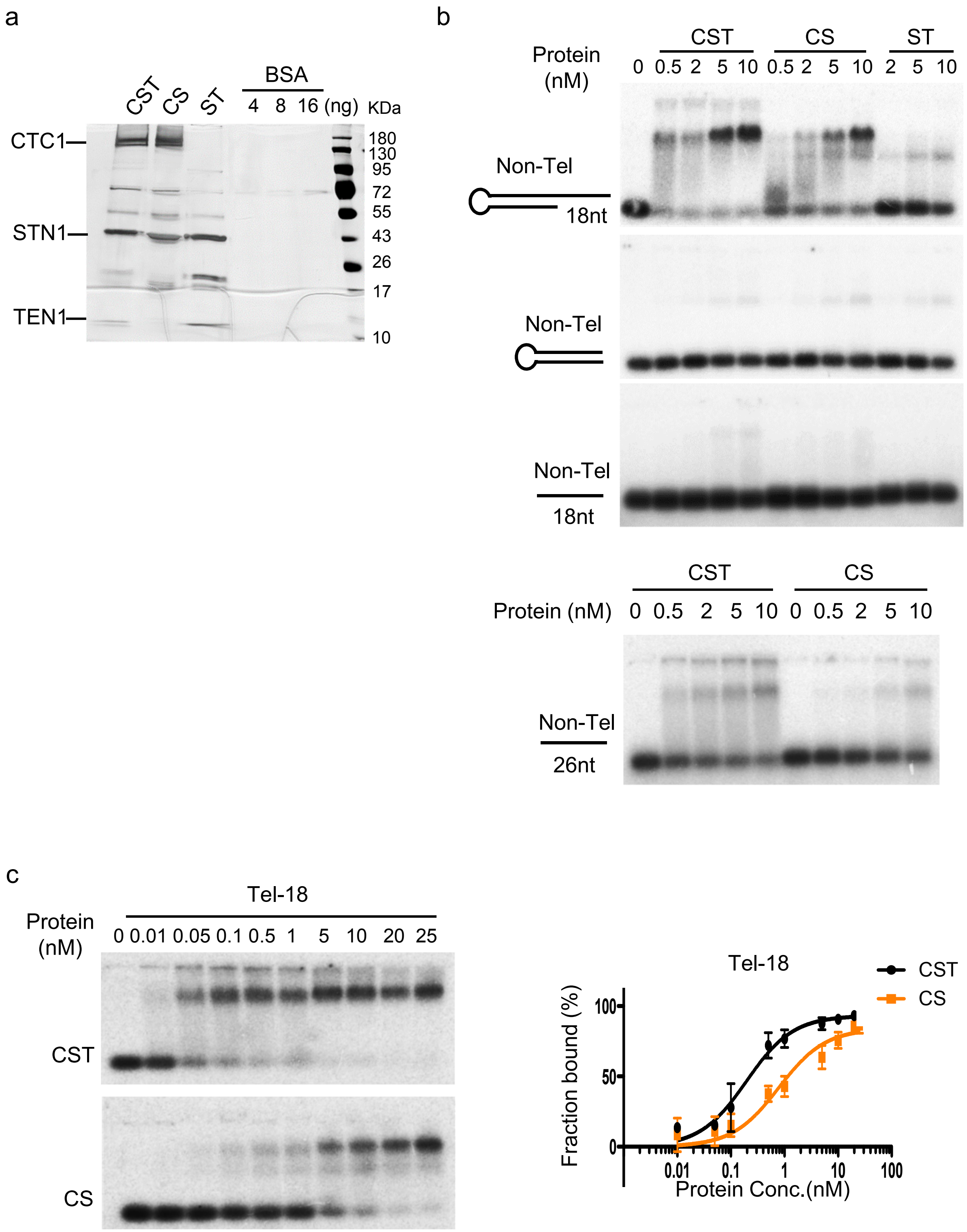
a
CST
CS
ST
BSA
4 8 16 (ng)
KDa
CTC1
STN1
TEN1
180
130
95
72
55
43
26
17
10
b
Protein (nM)
CST
CS
ST
0 0.5 2 5 10 0.5 2 5 10 2 5 10
Non-Tel
18nt
Non-Tel
Non-Tel
18nt
CST
CS
Protein (nM) 0 0.5 2 5 10 0 0.5 2 5 10
Non-Tel
26nt
c
Tel-18
Protein (nM)
0 0.01 0.05 0.1 0.5 1 5 10 20 25
CST
CS
Tel-18
CST
CS
Fraction bound (%)
100
50
0
0.01
0.1
1
10
100
Protein Conc.(nM)

d

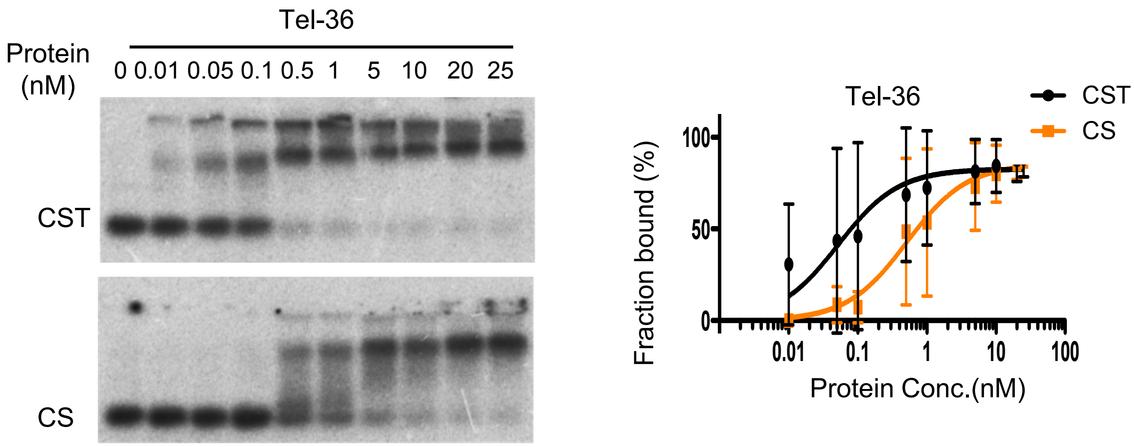
Tel-36
Protein (nM)
0 0.01 0.05 0.1 0.5 1 5 10 20 25
CST
CS
Tel-36
CST
CS
Fraction bound (%)
100
50
0
0.01
0.1
1
10
100
Protein Conc.(nM)

e

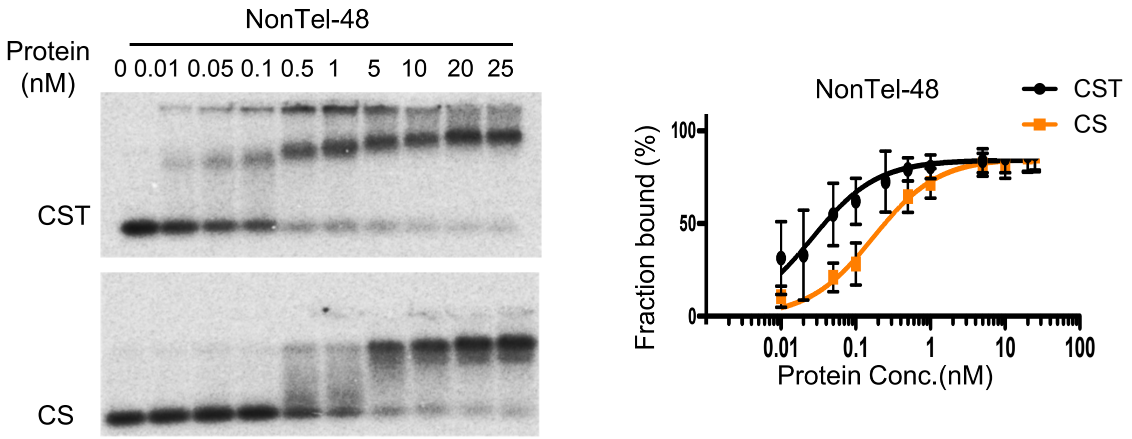
NonTel-48
Protein (nM)
0 0.01 0.05 0.1 0.5 1 5 10 20 25
CST
CS
NonTel-48
CST
CS
Fraction bound (%)
100
50
0
0.01
0.1
1
10
100
Protein Conc.(nM)

f

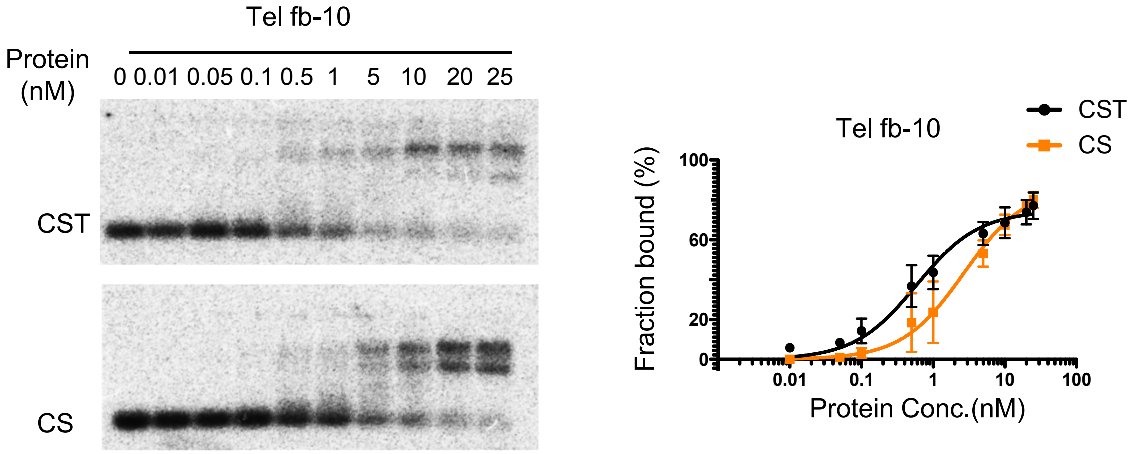
Tel fb-10
Protein (nM)
0 0.01 0.05 0.1 0.5 1 5 10 20 25
CST
CS
Tel fb-10
CST
CS
Fraction bound (%)
100
60
20
0
0.01
0.1
1
10
100
Protein Conc.(nM)

Supplementary Figure 5. CST, CS and ST affinity for ssDNA and ss-dsDNA junctions. (a) Silver-stained gel showing co-purification of CST, CS and ST. Combinations of FLAG-CTC1, FLAG-STN1 and TEN1 expression constructs were transfected into HEK293 cells, proteins were purified on FLAG beads. (b) EMSAs showing CST, CS or ST binding to non-telomeric ss-dsDNA junction substrate with 18 nt 3' overhang, same substrate lacking the overhang, 18 nt ssDNA of same sequence as the overhang or 26 nt non-telomeric DNA. (c-f) Examples of EMSAs (left) and corresponding plots (right) used to calculate Kd(apps) given in Fig 5B. Error bars indicate SEM.

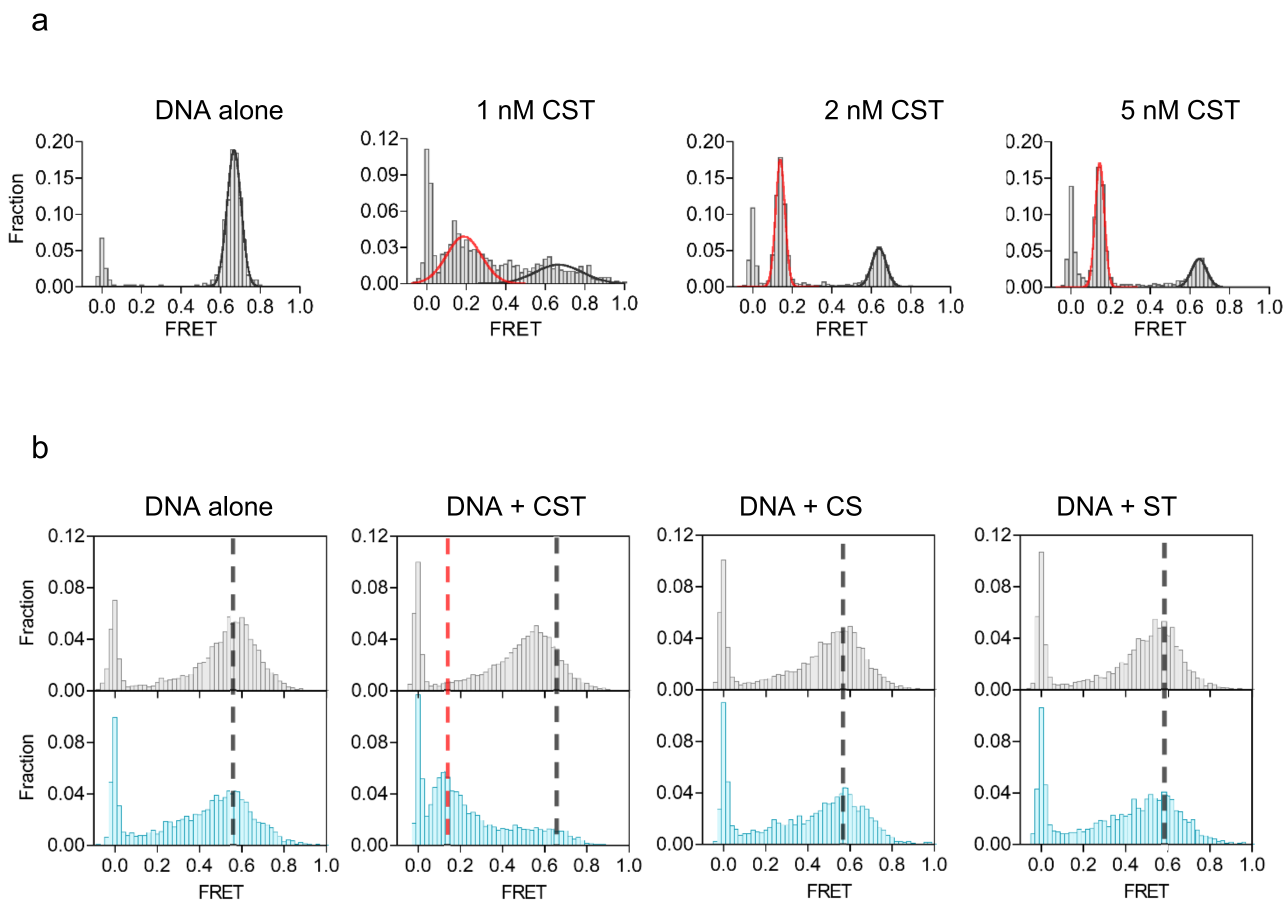

a
DNA alone
1 nM CST
2 nM CST
5 nM CST
Fraction
FRET
b
DNA alone
DNA + CST
DNA + CS
DNA + ST
Fraction
FRET

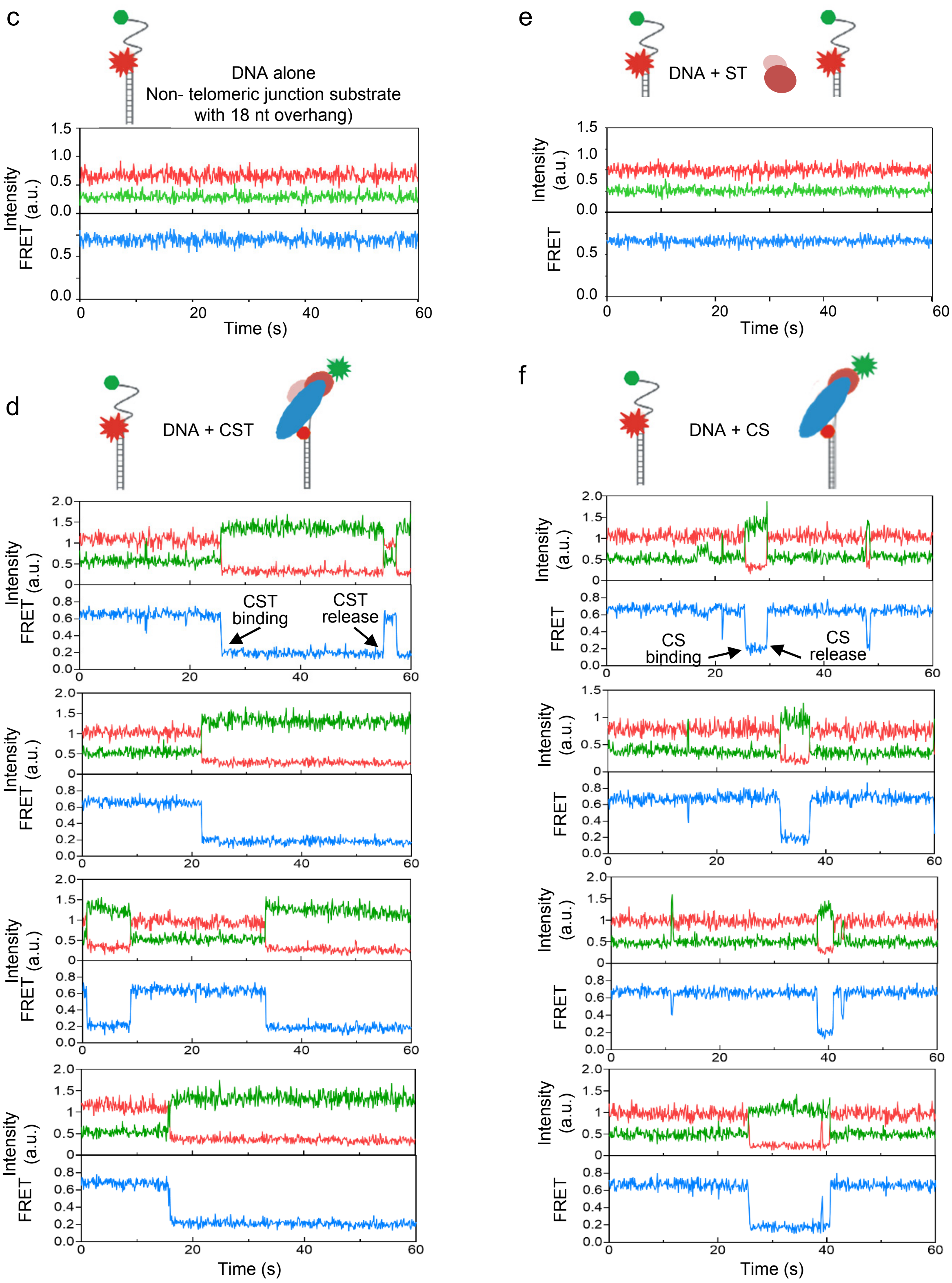

c
DNA alone
Non- telomeric junction substrate
with 18 nt overhang)
Intensity (a.u.)
FRET
Time (s)
e
DNA + ST
Intensity (a.u.)
FRET
Time (s)
d
DNA + CST
Intensity (a.u.)
FRET
CST binding
CST release
Time (s)
f
DNA + CS
Intensity (a.u.)
FRET
CS binding
CS release
Time (s)

g

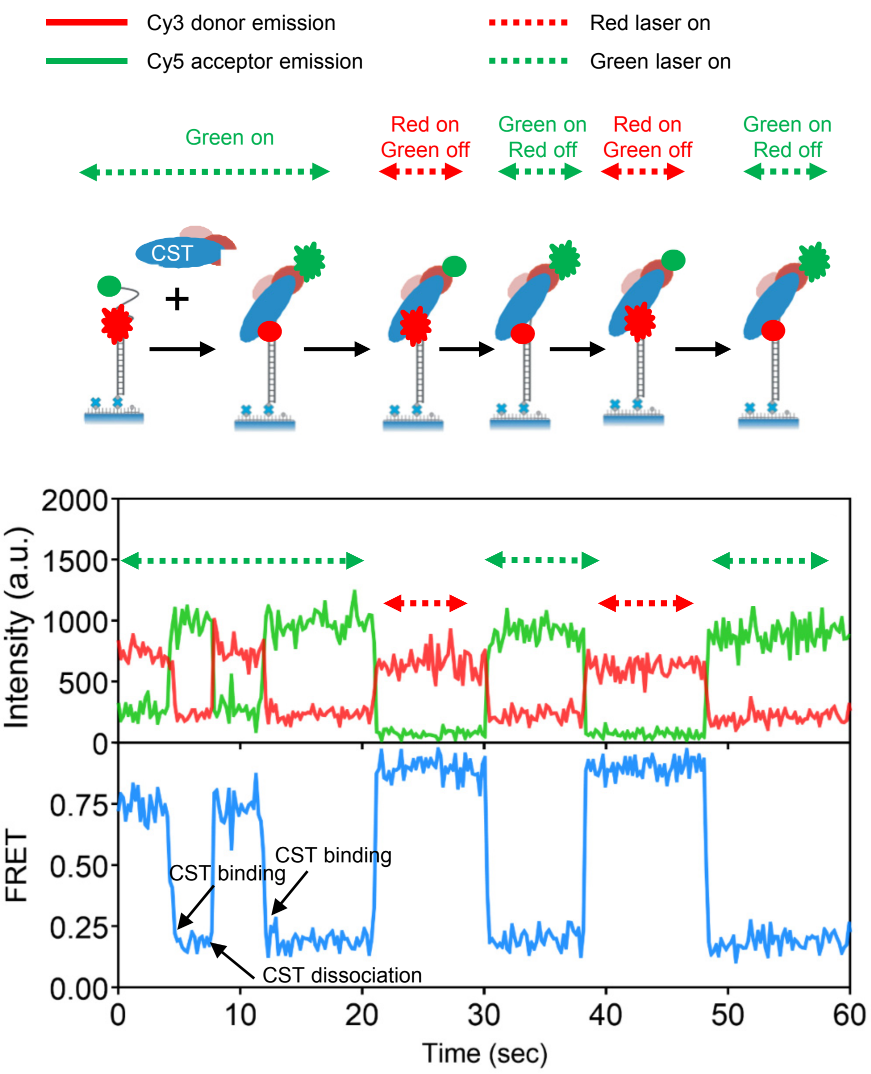

Cy3 donor emission
Cy5 acceptor emission
Red laser on
Green laser on
Green on
Red on
Green off
Green on
Red off
Red on
Green off
Green on
Red off
CST
+
2000
1500
1000
500
0
Intensity (a.u.)
0.75
0.50
0.25
0.00
FRET
CST binding
CST binding
CST dissociation
0
10
20
30
40
50
60
Time (sec)

Supplementary Figure 6. smFRET time-course traces showing CS, CST or ST binding to non- telomeric junction substrate. (a) FRET histograms showing that the fraction of substrate molecules giving a high FRET signal decreases with addition of increased concentration of CST to the flow chamber. CST remained in the chamber during data acquisition. FRET measurements were collected from >4,000 single molecules. (b) FRET histograms generated after CST, CS or ST washout. 2 nM protein was added to the flow chamber, incubated for 10 min then washed out with imaging buffer prior to data collection. (c-f)Traces show FRET (bottom) or individual Cy3 and Cy5 signals (top). Traces were recorded in the absence of added protein (c), after addition of 2 nM CST (d), ST (e), or CS (f). (g) Control experiment to show loss of FRET after CST addition reflects protein binding and not photobleaching. The slide was illuminated with the green laser (532 nm light) for ~20 sec (capturing two CST binding events and one CST dissociation event). The green laser was then turned off and the red laser (640 nm light) turned on to demonstrate that the Cy3 could still be excited by direct illumination. The switch in illumination was repeated several times to demonstrate that each dye remained active.

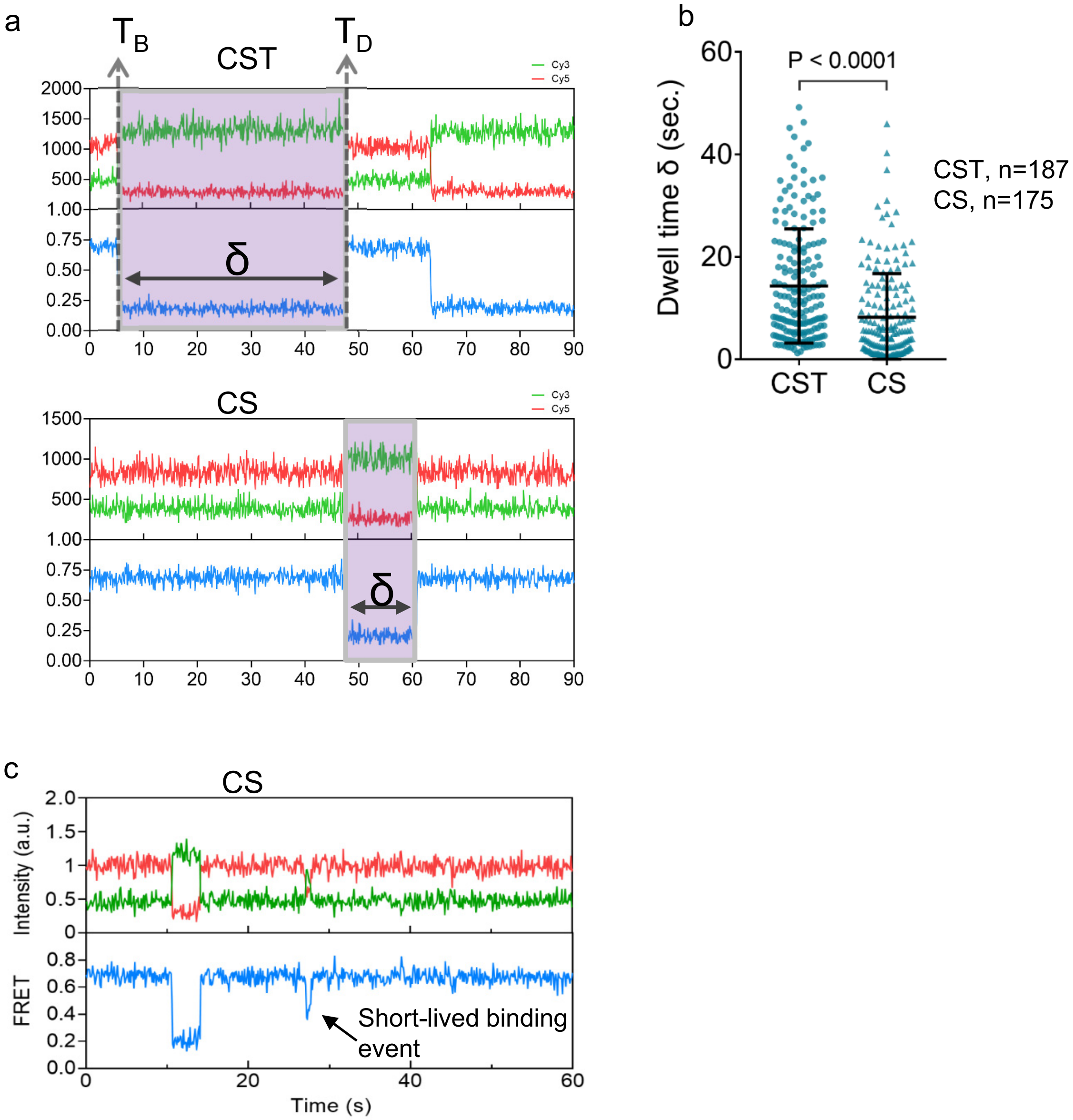

Supplementary Figure 7. Quantification of dwell times and transient binding events. (a) Examples of smFRET traces used to determine dwell times (δ) for CST or CS binding. The dwell time (δt) was taken as the time interval between protein binding and dissociation such that $\delta = ((T_D - T_B)$. A rapid decrease in FRET from 0.75 to ~0.15 was scored as a binding event while a sudden increase in FRET from 0.15 to 0.75 was scored as a dissociation event. The time interval between protein binding and release was calculated manually. Over 170 events were scored for each protein. (b) Dot plots showing average dwell well time for CST or CS binding before protein dissociation. Horizontal bars indicate mean and standard deviation. (c) smFRET trace illustrating transient binding events. These events were scored manually based on a decrease in FRET signal from 0.75 to 0.4-0.5. For each protein, >150 events were scored, all events had a dwell time of <3 sec. Precise dwell times were not determined..

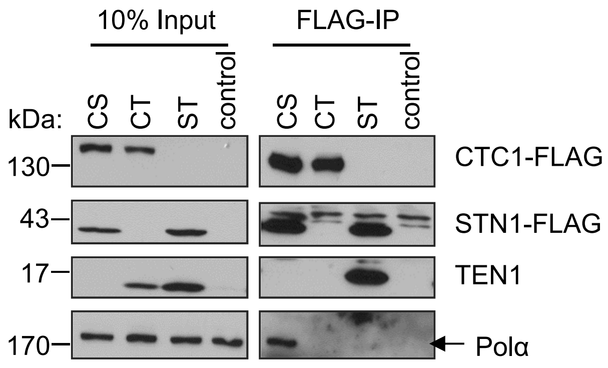

Supplementary Figure 8. Co-immunoprecipitation of DNA pol α with CS, CT and ST. FLAG-tagged CTC1/STN1 was precipitated HEK293 cell lysates using FLAG beads. Western blots were performed with antibody to FLAG, TEN1 or Pol α. The arrow indicates position of Pol α.

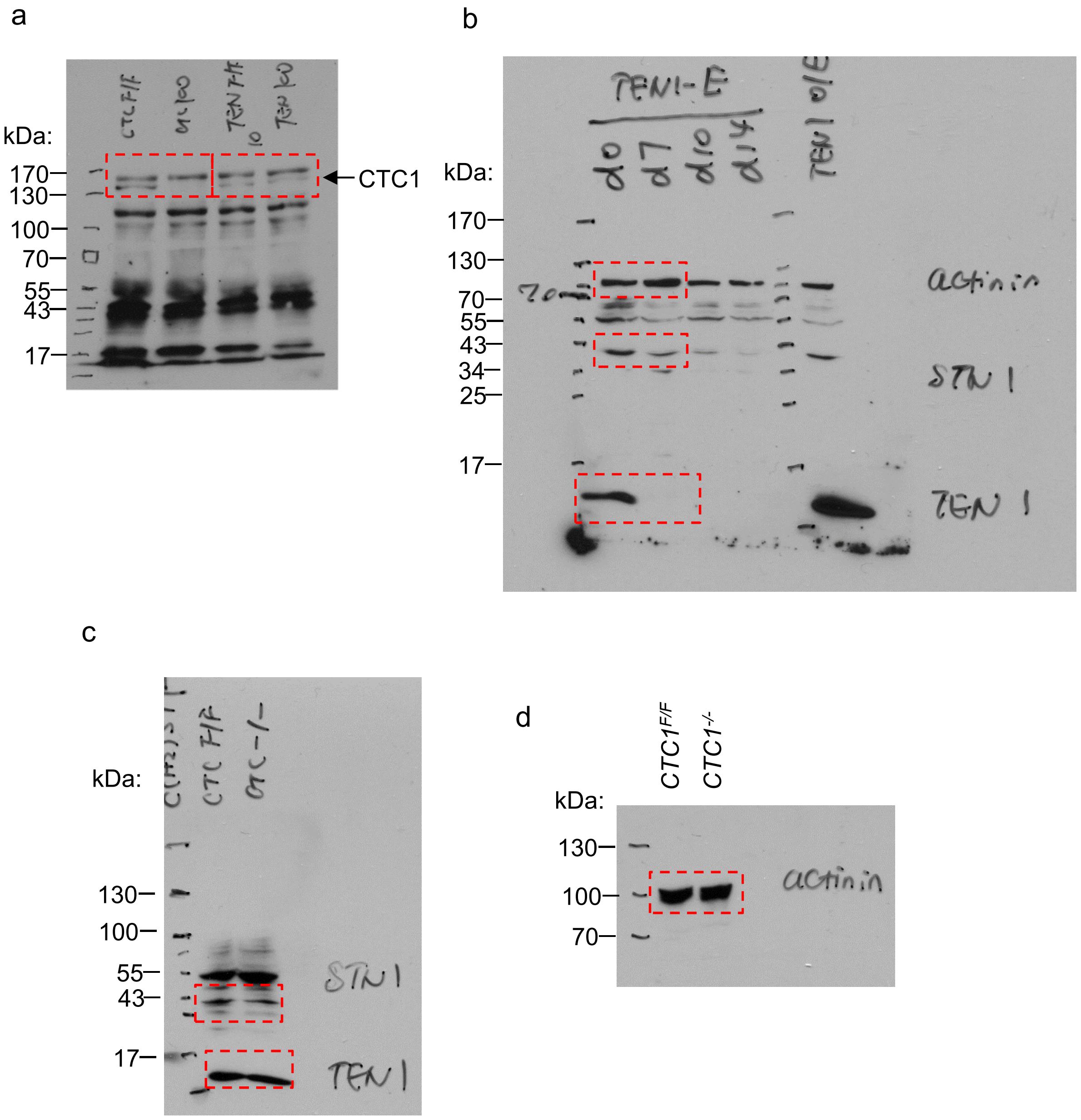

Supplementary Figure 9. The full blots showing western blot analysis of CST expression in TEN1 or CTC1 conditional knockout cells. (a) CTC1 expression. (b) STN1 and TEN1 expression in TEN1 knockout cells. (c) STN1 and TEN1 expression in CTC1 knockout cells. (d) Loading control for (c). Red boxes showing the cropped blots. a-b relate to Fig. 1c. c-d relate to Fig. 4. d-e.

Supplementary Table 1. Quantification of SFEs, sister chromatid association and chromosome fusion from metaphase spreads of CTC1 or TEN1 conditional cells prepared after various times of tamoxifen treatment

Days of TAM treatment	Signal free ends		Sister chromatid association		Chromosome fusions	
	$CTC1^{Cond}$	$TEN1^{Cond}$	$CTC1^{Cond}$	$TEN1^{Cond}$	$CTC1^{Cond}$	$TEN1^{Cond}$
Day 0	2.26%	1.60%	0.93%	0.82%	0.00%	0.00%
Day 7	3.58%	4.81%	2.10%	1.89%	0.07%	0.35%
Day 10	4.10%	5.41%	2.32%	0.96%	0.05%	0.33%
Day 14	6.04%	5.46%	1.51%	1.83%	0.12%	0.37%

Values represent % of chromosomes with one or more SFE or associated sisters. Chromosome fusions (chromosome or chromatid) were scored for number of fusions per 100 chromosomes. N = 3 experiments, 2,000 chromosomes were scored per time point.

Supplementary Table 2. Quantification of ChIP data from Fig. 4 and Supplemental Fig. 4

		Repeat 1	Repeat 2	Repeat 3	Average	Std. dev	T test
STN1-ChIP	CTC1F/F	0.0373	0.0321	0.0338	0.0344	0.00263	
	CTC1-/-	0.00711	0.00997	0.0158	0.0110	0.00443	0.00317
	TEN1F/F	0.0498	0.0402	0.0565	0.0488	0.00822	
	TEN1-/-	0.0756	0.0533	0.0630	0.0640	0.0112	0.137
CTC1-ChIP	TEN1 F/F	0.119	0.102	0.091	0.104	0.0139	
	TEN1 -/-	0.105	0.211	0.221	0.179	0.0642	0.174
TEN1-ChIP	CTC1 F/F	0.143	0.090	0.106	0.113	0.0270	
	CTC1-/-	0.0331	0.0322	0.0472	0.0375	0.00840	0.0310

Supplementary Table 3. Total number of scored short and long-lived CS binding events

18 nt + CS			
	Number full/long-lived binding & dissociation events (FRET 0.75 → ~0.15 → 0.75)	Number short-lived binding events (FRET 0.75 → ~0.4 → 0.75)	Fraction short versus long-lived events
Exp. 1	249	53	0.2129
Exp. 2	179	46	0.2570
Exp. 3	203	44	0.2167
TOTAL	631	143	**0.2266**

Supplementary Table 4: Primers and oligonucleotides used in cell line generation and RT-PCR

**Primer/ Oligo**	**5'→3**
TEN1 intron 2 LoxP FP	CTGAAATGTCTCAAGTAAACAGCAG
TEN1 intron 2 LoxP RP	ATGAGCCACCACACCTGATC
TEN1 intron 3 LoxP FP	ACTCAAAGACAGGGTGGCTG
TEN1 intron 3 LoxP RP	GATGGAGGTTGCAGTGAGCT
TEN1 intron 2 LoxP donor oligonucleotide	ACCTATAGTCCCAGCTACTCAGGAGGCTGAGA<br>TGGGAGGATTGCTTGAGCAGCCTGGAAGGCATGCATAA CTTCGTATAGCATACATTATACGAAGTTATTCAAGGCTGC AGCCTGGGATACAGAAAAAAAAAAAAAAAGAAAGGAAGAA AAAGATTTCTTAGACAAGACATAGAAAGCAAAAGTCA
TEN1 intron 3 LoxP donor oligonucleotide	ACTGCACTCCAGCCTAGATGACAGAGTGAGACTCCGTCT CAAAAAAAAAAAAAAAAAAAAAAGACAGGTACAGTGAAG AGACAATCCCGCTATGCATAACTTCGTATAGCATACATTA TACGAAGTTATCCATGGGGAAGTGAGAACGAGGAGAGA GAAACCACTAATTTCTCAAGGCCTACAACACAC
P53 RT-PCR FP	GCCCAACAACACCAGCTCCT
P53 RT-PCR RP	CCTGGGCATCCTTGAGTTCC
hTERT RT-PCR FP	GGTGGATGATTTCTTGT
hTERT RT-PCR RP	GTGAAACCTGTACGCC
β-actin RT-PCR FP	GCTCCGGCATGTGCAAGG
β-actin RT-PCR RP	ATGAGGTAGTCAGTCAGGTC

Supplementary Table 5: Oligonucleotides used for EMSA and smFRET

**EMSA**	**5'→3'**
Tel-18	(GGTTAG)$_3$
Tel-36	(GGTTAG)$_6$
NonTel-48	AGCGTATCCGTTCAGTTGAGCGTATCCGTTCAGTT<br>GAGCGTATCCGTT
Tel fold-back 10 nt 3' overhang	CCTACTGCATCTAGCTTTTTGCTAGATGCAGTAGG<br>TTAGGGTTAG
Tel fold-back blunt	CCTACTGCATCTAGCTTTTTGCTAGATGCAGTAGG
Tel10	TTAGGGTTAG
NonTel fold-back 18 nt 3' overhang	ACGACTGCATCTAGCTTTTTGCTAGATGCAGTCGT<br>AGCGTATCCGTTCAGTTG
NonTel fold-back blunt	ACGACTGCATCTAGCTTTTTGCTAGATGCAGTCGT
NonTel-18	AGCGTATCCGTTCAGTTG
NonTel-26	GCGTACTCAGTGACTCAGATCGAC
**smFRET**	**5'→3'**
Cy5 18 nt anchor	Cy5-GCCTCGCTGCCGTCGCCA-Bio
Cy3 18 nt overhang	TGGCGACGGCAGCGAGGCAGCGTATCCGTTCAGT<br>TG-Cy3

Underline indicates regions of dsDNA
